# 

**Published:** 1887-05

**Authors:** 


### AMERICAN JOURNAL

—OF—

### DENTAL SCIENCE,

—EDITED BY—

F. J. S. GORGAS, M. D., D D. S.

---

VOLUME XXI—Third Series.

---

1887–88.

SURGEON GEN'L'S OFFICE
LIBRARY.
115685

BALTIMORE:

SNOWDEN & COWMAN,

LONDON:

TRUBNER & CO. 60 PATERNOSTER ROW,

1888.

### Index to Volume XXI.---Third Series.

A Factor in Tooth Preservation, - - - 1
Alveolar Abscess, - - - - - 21
A Method of Filling very Fine Root Canals, - 45
A Method of Bridge-Work, - - - - 46
American Dental Association, - - 87, 89, 139
A Romantic History Connected with a Prominent Dentist, 90
Artificial Teeth, - - - - - 93
Amalgam, - - - - - - 96
Antiseptics and Disinfectants in Office Practice, - 104
Amalgam and Matrix, - - - - - - 126
A Method of Testing the Vitality of a Tooth's Pulp, 141
Anæsthetics and Anæsthesia, - - - - 218
A Case of Replantation, - - - - 239
Antisepsis, - - - - - - 306
A Visit to Foreign Dental Schools and other Observations, 320
Are there too Many Dentists? - - - - 324
An Artificial Crown, - - - - - 326
A Dentist's Bill, - - - - - 336
American Dental Society of Europe, - - 360
A New Method of Treating Teeth with Diseased Pulps, 399
A Case of Somewhat Remarkable Character, - 432
A Receipt for Mending Broken Marble, - - 479
A very Valuable Lesson for those who Use Anæsthetics, 494
A Misplaced Right Superior Cuspid Tooth with Resulting Sequelæ, - - - - - 510
Ammonia vs. Zinc Fillings, - - - - 515
A Voice Raised Against Carpets, - - - 522
Anchor Pins, - - - - - - 525
Against Indiscriminate Use of Tea and Coffee, - 528
A Cheap Matrix, - - - - - - 572
A Physician and the Tooth-ache, - - - 576
Annual Commencement of the Baltimore College of Dental Surgery, - - - - - - 569
Annual Commencement of the Dental Department of University of Maryland, - - - 571
British Dental Association, - - - - 260
Bleaching Teeth, - - - - - - 47
Blind Abscess, - - - - - - - 115
Bridge-work and Tooth Crown Patents, - - 426

BIBLIOGRAPHICAL.
A System of Dental Surgery, - - - . 44
The American System of Dentistry - - 287
Holden's Anatomy, - - - - 288
Transactions of the Illinois State Dental Society, 288
Dental Chemistry—The Dentist's Manual of Special Chemistry, - - - - - - 474
Memoirs of a Southern Planter, - - 475
Irregularities of the Teeth and their Treatment, - 476
Vick's Floral Guide, - - - - 476
Nitrous Oxide—Its Properties—Methods of Administration and Effects, - - - 477
The Physician's Visiting List, 1888, - - 477
Pearson's Dentist's Appointment Book - 477
Transactions of the American Dental Association, 477
Clamp Plate, - - - - - - 118
Composition for Duplicating Models, - - - 143
Capping Exposed Pulps, - - - - 289
Cases in Practice, - - - - - 328
Chinese Anæsthetic, - - - - - 478
Cleaning Teeth, - - - - - - 458
Cocaine and Carbolic Acid Anæsthesia, - - 526
Conservation of the Dental Pulp, - - - - 554
Daily Life in a Dental Infirmary, - - - 39
Dentistry Recognized by the American Dental Associatiation as a Specialty of Medicine, - - 143-236
Death in a Dental Office, - - - - 232
Dental Ethics, - - - - - - 275
Dental Department of the University of Maryland, - 332
Death During Ether Anæsthesia, - - - 335
Difference Between Creosote and Carbolic acid, - 480
Dark Joints, - - - - - - - 520
Death of a Lady after having Seventeen Teeth Extracted, 336
Electricity and its Application to Dentistry, - 14
Extract from a Monograph on Local Anæsthesia in Dentistry and Minor Surgery, - - - 30
Elements, - - - - - - - 92
Extracts from Lectures on Operative Dental Surgery, 120
Epileptiform Neuralgia of the Fifth Nerve, - - 145

Erythroxylon Coca and its Alkaloid Cocaine, - 177
Educated Ability and Industry Essential to Success in the Practice of Dentistry as a specialty, - - 209
Effects of Study on the Teeth, - - - 336
Experience of an English Dentist who had Two Teeth Implanted, - - - - - - 382
Examination Questions—Royal College of Surgeons of England, - - - - - - 384
Extracts from Lectures on Operative Dental Surgery, 469
Experiments, - - - - - - 523
Florida Dental Law, - - - - 227
Failures of Fillings, - - - - - 447
Flies in the Operating Room, - - - 524
Gold Solder, - - - - - - 144
Gold at Cervical Border, - - - - 229
Gold is and will Remain the King of Filling Material, 524
Hypnotic Experiments in Paris, - - - 238
Hours of Fate, - - - - - - 480
How to Cut a Bottle. - - - - 574
Implantation, - - - - - - 24
Immediate Root Filling, - - - - 134, 537
Improved Artificial Teeth, - - - - 402
In Regard to Obtundents, - - - - - 528
Intermittent Pressure—Its Relation to Orthodontia, - 563
Irregularities, - - - - - 575
Indirect and Improper Advertising, - - - 332
International Medical Congress, - 182, 184, 241, 232
Laboratory Hints, - - - - - 94
Lectures on Dentistry for Popular Audiences, - 109
Lodgment of a Tooth Plate in the Gullet for Fifteen Months, - - - - - - 431
Lubricating Jack Screws, - - - - 528
Lead or Amalgam Instead of Gold, - - 573
Metal Work, - - - - - - 35
Missouri State Dental Association, - - - 140
Management of Children's Teeth, - - - 206
Myrtol, - - - - - - 240
Mastication, - - - - - - 480
Mercurial Paralysis, - - - - - 480

North Carolina's Amended Dental Law, - - 285
Odonto Chirurgical Society of Scotland, - - 76
Observations and Experiments on the Action of Certain Anæsthetics, Alone or Mixed, on the Fibres of the Fifth Pair of Nerves, - - - - 129
Obtunding Sensibility, - - - - 144
One Objection to Plastic Fillings, - - - 235
Operative Dentistry as applied to Deciduous Teeth, 312
Odontomes, - - - - - - 405
On the Combination of Tin and Gold as a Filling Material For the Teeth, - - - - - 418
On the Curability of Pulpless and Abscessed Teeth; Mainly by the Immediate Method, with Statistics of Cases, - - - - - - 433
On the Causes of Acute Suppurations in the Light of Present Scientific Views, - - - 517
Odonto-Chirurgical Society, - - - - 529
On Some Cases of Congenital Fissures of the Mouth, 549
OBITUARY.
Dr. J. R. Walker, - - - - - 190
Ralph C. Purnell, M. D., D. D. S., - - 190
John McCalla, D. D. S., - - - - 287
D. M. Parker, D. D. S., - - - 333
Hugh McGinnis Grant, D. D. S., - - - 428
Pathology of Dental Caries, - - - 112
Proximal Decays, - - - - - - 141
Painless Extinction of Life in the Lower Animals, - 142
Pulse in Morphiomania, - - - - - 240
Pulpless Teeth and their Treatment, - - 294
Pure Mercury, - - - - - - 478
Perseverance an Important Factor, - - 521
Poisoning with Iodol, - - - - - 527
Resolution of American Medical Association, - 225
Root Medication, - - - - - - 545
Southern Dental Association, - 89, 185, 231, 337, 385
Students' Society, - - - - - - 95
Spiders, - - - - - - - 239
Soft Gold Foil, - - - - - 375
Sensitive Dentine, - - - - - - 447

The Matrix in Filling Teeth, - - - - - 37
The Value of a Name in Dentistry, - - 43
Teething. - - - - - - - 48
The Odontological Society of Great Britain, - 49
The National Association of Dental Examiners, - 89
The Types of Teeth in Disease, - - - 97
The Importance of a Liberal Professional Education in the Practice of Dental and Oral Surgery, - 159
The Preparation of Tissues for Examination with the Microscope with Special Reference to the Microscopical Examination of the Teeth, - - - 170
Temperature of the Human Skin, - - - 191
Teething: Is it a Common Cause of Disorder? - 193
Toxic Effects of Lead, - - - - - 235
Treatment of Diarrhœa by Iodoform and Charcoal, 237
The Adulteration of Candies, - - - - 240
The Abuse of Dental Charity, - - - 281
The Six Year Molars, - - - - - - 302
The Use of the Microscope in Progressive Dentistry, 316
The First District Dental Society of the State of New York, 331
Trophic Nerves, - - - - - - 334
Two Cases of Epulis, - - - - - 430
The Immediate Filling of Pulpless Teeth, - - 460
The Etiology of Caries of the Teeth Viewed from the Standpoint of Physiological Chemistry, - 464
The Control of Hæmorrhage, - - - - 479
The Pathology of Alveolar Abscess, - - 481
The Application of Herbst's Method to Tin-Gold Fillings, 519
Thorough Examination, - - - - 522
The Cultivation of the Faculty of Knowing, - - 525
Talent vs. Genius, - - - - - - 526
Tumors of the Mouth and Jaws, - - - 558
The Most Convenient Matrix, - - - 576
Uses of Chlora-Percha, - - - - 575
Virginia State Dental Association, - - - 88
Vacuum Plates, - - - - - - 299
What is Pain? - - - - - - 523
Water at Meals, - - - - - - 574

### *To Readers and Correspondents!*

Communications intended for publication, and exchange journals and papers should be addressed to the EDITOR OF THE AMERICAN JOURNAL OF DENTAL SCIENCE, 845 N. Eutaw St., Baltimore, Md. All letters relating to the business of the JOURNAL, or containing cash remittances, to be directed to Messrs. SNOWDEN & COWMAN. Articles for publication should be received by the editor at least three weeks previous to the first month on which the number in which it is designed for them to appear, is issued.

CONTENTS:

ARTICLE I.—A Factor in Tooth Preservation. By Prof. C. N. Pierce..... 1

" II.—Electricity and its Application to Dentistry. By Dr. George C. Brown..... 14

" III.—Alveolar Abscess..... 21

" IV.—Implantation..... 24

" V.—Extract from a Monograph on Local Anæsthesia in Dentistry and Minor Surgery. By. Prof. George Viau; translated by L. Turnbull, M. D .. 30

" VI—Metal Work. By Dr. L. P. Haskell..... 35

" VII.—The Matrix in Filling Teeth..... 37

EDITORIAL, ETC.

Daily Life in a Dental Infirmary..... 39
The Value of a Name in Dentistry..... 43

BIBLIOGRAPHICAL.

A System of Dental Surgery..... 44

MONTHLY SUMMARY.

A Method of Filling Very Fine Root Canals..... 45
A Method of Bridge-Work..... 46
Bleaching Teeth..... 47
Teething..... 48

☞ The AMERICAN JOURNAL OF DENTAL SCIENCE will contain fifty pages of reading matter in each number, making a volume of about *Six Hundred* pages,

EXCLUSIVE OF ADVERTISEMENTS.

